# High regional variability of HIV, HCV and injecting risks among people who inject drugs in Poland: comparing a cross-sectional bio-behavioural study with case-based surveillance

**DOI:** 10.1186/s12879-015-0828-9

**Published:** 2015-02-21

**Authors:** Magdalena Rosińska, Janusz Sierosławski, Lucas Wiessing

**Affiliations:** Department of Epidemiology, National Institute of Public Health, National Institute of Hygiene, Chocimska 24, 00-791 Warsaw, Poland; Institute of Psychiatry and Neurology, Sobieskiego 9, 02-957 Warsaw, Poland; European Monitoring Centre for Drugs and Drug Addiction (EMCDDA), Cais do Sodré, 1249-289 Lisbon, Portugal

**Keywords:** People who inject drugs, Injecting drug use, HCV, HIV, Prevalence, Surveillance

## Abstract

**Background:**

People who inject drugs (PWID) are an important group at risk of blood borne infections in Poland. However, robust evidence regarding the magnitude of the problem and geographical variation is lacking, while coverage of prevention remains low. We assessed the potential of combining bio-behavioural studies and case-based surveillance of PWID to gain insight into preventive needs in Poland.

**Methods:**

Results of a bio-behavioural human immunodeficiency virus (HIV) and hepatitis C virus (HCV) prevalence study among ever injectors in six regions in Poland were compared with HIV case-based surveillance trends from 2000 to 2012. Logistic regression was used for multivariable analyses in the prevalence study. The case surveillance data were correlated with prevalence data, by region, to determine surveillance validity and identify any recent trends.

**Results:**

HIV seroprevalence (18% overall) differed more than ten-fold across regions (2.4% to 32%), but HCV seroprevalence and the proportion of PWID sharing needles/syringes in the past 12 months were similar, 44% to 68% and 22% to 29%, respectively. In multivariable models accounting for socio-demographic factors, duration of injecting history and needle sharing practices, regional differences were significant for both HIV and HCV seroprevalence with adjusted odds ratios varying up to a factor of 12.6 for HIV and 3.8 for HCV. The number of new cases of HIV diagnosed in each region during the bio-behavioural study period was strongly correlated (r = 0.93) with HIV prevalence. There was an overall decreasing trend in the number of new diagnoses of HIV over time. However, a transient increase in three regions was preceded by a higher proportion of people with short injecting history (≤5 years) and a high prevalence of HCV coinciding with a low prevalence of HIV in the bio-behavioural study.

**Conclusions:**

Bio-behavioural and case-based data were consistent with respect to the regional distribution of HIV and also provided complementary information, with the proportion of new injectors and high HCV prevalence predicting increases in HIV case rates. We identified three regions in Poland that appear to be at increased need for preventive measures. Data point to the need for a stronger investment in harm reduction programmes in Poland.

**Electronic supplementary material:**

The online version of this article (doi:10.1186/s12879-015-0828-9) contains supplementary material, which is available to authorized users.

## Background

Injecting drug use is one of the key risk behaviours associated with transmission of blood borne viruses such as human immunodeficiency virus (HIV) and hepatitis C virus (HCV) [1-3]. In regions of Eastern Europe bordering the European Union (EU), the HIV epidemic is still driven primarily by injecting drug use. In the EU, although the trend in HIV cases related to injecting drug use has been downward for over a decade, large outbreaks of HIV among people who inject drugs (PWID) have recently occurred in countries where HIV transmission among PWID had remained low, suggesting that ongoing surveillance is of key importance [4-9].

Public health interventions aimed at reducing high-risk drug injections, such as provision of clean supplies (needle/syringe programmes) and, most importantly, opioid substitution therapy, have proven effective in curbing HIV infections among PWID [10-15]. The evidence for these interventions for HCV is weaker. For these measures to be fully effective, especially in the case of HCV, they must be implemented in a large scale and with high coverage. Insufficient coverage or limited access in some areas could potentially hamper the impact of these interventions on reducing blood borne infections [1,16]. Large differences in the availability of preventive programmes have been noted between Eastern and Western Europe (the EU), which are associated with the diverging epidemic trends seen between these regions [17,18]; the recent outbreaks of blood borne infections in the EU have also been associated with low and/or interrupted coverage of harm reduction programmes [5-9].

In Poland, public health interventions introduced in 1989 and 1990 in response to the initial HIV epidemic among PWID were based on the principle of reducing risks associated with injecting drug use. However, availability of harm reduction programmes has remained low. Opioid substitution programmes had space for just over 1,500 patients in 2009 for an estimated 25,000 to 29,000 problem opiate users. In 2011, space was available for 2,200 patients for 10,400 to 19,800 problem opiate users, suggesting a coverage of 5% to 15% [19,20]. Approximately 69% of opioid users entering treatment programmes inject drugs, suggesting some 17,000 to 20,000 opioid injectors in 2008 and 7,200 to 13,700 in 2011. Still, of nearly 90 low-threshold services for PWID available in 2008, only 27 had needle/syringe distribution or exchange programmes. Approximately 430,000 needles were distributed or an estimated 20 to 25 needles per opioid injector [21], not including persons injecting other substances. In 2011, the number of syringes distributed declined even further, to approximately 195,000, or between 14 and 27 syringes per opioid injector.

Continued transmission of blood borne infections among PWID in Poland is evidenced by surveillance data. Approximately 18% of newly diagnosed cases of HIV infection, where the mode of transmission was reported, during 2008 to 2011 and 11% of reported cases of acute HCV infection during 2009 to 2011 were attributed to PWID (National Institute of Public Health – National Institute of Hygiene, unpublished data). In view of the increasing numbers of newly diagnosed HIV cases, a re-evaluation of the role of various subpopulations, including PWID, in perpetuating the blood borne infections epidemics in Poland is needed. However, case-based reporting is affected by a number of factors, such as the frequency and patterns of testing for these infections. Moreover, no behavioural information is collected through this system, making it insufficient to guide policy decisions at national and regional levels. Ideally, repeated bio-behavioural studies should be the backbone of surveillance [22,23]. However, in most countries these studies are regarded as too resource-intensive and are not conducted on regular basis [24].

The last large prevalence study in Poland that did collect behavioural information was performed from 2004 to 2005. Although there were no abrupt changes in either the pattern of drug use or in the public health response system, including the provision of harm reduction services, the time elapsed since data collection may affect the relevance of the results of this survey to recent policies. We therefore aimed to assess the potential of combining and cross-validating bio-behavioural studies and case-based surveillance among PWID, to understand underlying risk behaviours and the composition of the PWID population regarding characteristics such as age, preferred drug use, and history of injecting drug use. In particular the aim was to identify factors that may be associated with trends in HIV reporting rates.

## Methods

We performed descriptive analyses to compare the outcomes of a bio-behavioural study among PWID to the trends observed in case-based HIV surveillance data for PWID from 2000 to 2012 at the regional level. All available records in the bio-behavioural dataset were included in the analysis. However, case-based surveillance data were limited to the cases residing in the regions included also in the bio-behavioural study at the time of HIV diagnosis. The datasets are described in the following sections. Modelling studies suggest that the interrelationship between HCV and HIV prevalence may be used to predict future HIV risk. Sites or regions where high HCV prevalence co-exists with low HIV prevalence among PWID may be at increased risk for an HIV outbreak [25,26]. We considered the following factors from the bio-behavioural study by region: HIV prevalence, HCV prevalence, adjusted odds ratios (OR) of HIV/HCV infections between regions, sites with ratio of HCV to HIV prevalence higher than average, the proportion of short-term injectors (<5-year history), the proportion of opioid users and the proportion sharing needles/syringes within the past 12 months. All analyses were performed using STATA version 13.1.

### Bio-behavioural study

#### Study design and participants

A cross-sectional bio-behavioural survey was carried out from 2004 to 2005 at 15 sites in 6 regions of Poland (Mazowieckie [Warszawa], Lubuskie [Zielona Góra, Gorzów Wielkopolski, Cibórz, Nowy Dworek], Śląskie [Katowice, Chorzów, Sosnowiec], Dolnośląskie [Wrocław, 2 sites], Lubelskie [Lublin, Puławy], Warmińsko-mazurskie [Olsztyn, Elbląg, Barczewo]). A convenience sample was obtained at each site using a snow-ball approach for recruitment from streets and low-threshold facilities and an exhaustive sampling from inpatient treatment centres (including detoxification wards and long-term drug-free treatment centres). All subjects meeting inclusion criteria of having injected drugs at least once during their lifetime (self-reported), residing in the regions of interest for at least 3 months prior to the study and over 18 years of age were invited to participate in the study. Non-response was not recorded. Each individual who gave informed consent was asked to fill out a questionnaire and provide a sample of venous blood for testing. Details of the study design have been reported elsewhere [27]. The study was approved by the Ethical Committee of the National Institute of Public Health – National Institute of Hygiene in Warsaw.

#### Laboratory analysis

Serum samples were tested for anti-HIV (HIV-Ab) and anti-HCV antibodies (HCV-Ab) using commercial testing kits with comparable parameters [28,29]. Enzyme-linked immunoassay (ELISA) was used to obtain HIV-Ab status (AxSym Abbott HIV 1/2 or Viranostika Organon-Teknika). All samples were tested twice, and the samples with positive results for both tests were considered positive. The samples with negative results in both tests were considered negative. HIV status was considered undetermined otherwise. A third generation HCV test was used to test for total HCV-Ab (AxSym Abbott HCV version 3.0, UBI HCV EIA 4.0 – Organon Technika).

#### Variable definition and statistical analysis

Needle sharing was defined as receptive sharing (i.e. using the same needles together with another person or using needles or syringes already used by another person). Information on sharing of other equipment, excluding needles and syringes, was included as an additional risk. Current employment status was defined as the primary occupation at the time of survey or before admission to an inpatient treatment; homelessness was having to live on the street or at a shelter at any time in the past 12 months and any time ever; and imprisonment was being in police custody or serving a prison sentence at any time in the past 12 months or any time ever. The chi-square test was used for univariable comparisons of categorical variables. Logistic regression was used for multivariable comparisons with backward deletion from first step models including variables significant at 0.1 level in the univariable analysis. Cases with missing values were excluded from analysis. Recruitment setting and duration of injecting drug history were forced into all models to avoid potential confounding. In case of categorical variables with more than two categories, if some categories were not significantly different at 0.05 level in multivariable models, these categories were collapsed. The factors not significant at 0.05 level and not confounding other factors were removed from the models. After selecting satisfactory models for HIV and HCV risk (second step models), final models were constructed by including all variables appearing in either of the second step models for greater comparability of HIV and HCV predictors.

### Case-based surveillance data

We extracted data on HIV cases diagnosed from 2000 to 2012 and reported to the national surveillance system by June 2013. The surveillance system is organised uniformly throughout the country and was stable in the examined time period. Reporting diagnosed cases of HIV infection to regional public health departments is mandatory for both clinicians and laboratories. Name-based identifiers are used. The HIV case definition requires confirmation of HIV infection by immunoblot or nucleic acid testing. Epidemiologists at the regional level verify case definitions, remove duplicates and forward the reports to the central level, where the process is repeated before entering data into the national database.

Clinical and epidemiological information (e.g., transmission category) is usually not available at the reporting laboratories. Additionally, case ascertainment by clinicians’ reports is lower in comparison to laboratory reporting, leading to a high proportion of missing data on clinical characteristics and transmission category. In order to determine trends in the number of cases attributed to injecting drugs, we performed multiple imputations of transmission category and region with iterative chained equations, using multinomial logistic regression models (with explanatory variables as year of diagnosis, age, sex, urban or rural residence, region and transmission category) for both imputed variables [30,31]. The number of cases by year and region was estimated using the Little-Rubin rule for regions where the prevalence study was conducted [30]. Poisson regression was used to evaluate trends in the number of cases by region.

## Results

### Group characteristics–bio-behavioural study

A total of 776 PWID were recruited into the study; 512 (66%) of these participants were outside of inpatient treatment centres. Valid test results for both HIV and HCV infections were obtained for 763 (98%) study participants, and the remaining 13 respondents were excluded from the analysis. After these cases were excluded, there were 217 (29%) females and 540 (71%) males, with mean and median ages of 29 and 26 years, respectively (see Additional file 1).

New injectors (first injection <2 years prior to the interview) and recent injectors (within the past 30 days) comprised 18% and 82% of the study population, respectively. The majority of respondents were recent opioid users (within the past 30 days). The most common drug of choice in the past 30 days was heroin (42%) followed by homemade opiates (23%) and amphetamines (20%). In total, 599 (82%) respondents had injected drugs in the past 30 days, including 283 (89%) heroin users, 174 (100%) homemade opiate users and 103 (72%) amphetamine users. Almost half of the respondents had injected with used needles that they reported as ‘most often’ not being disinfected. A significant proportion (23%) had ‘most often’ shared drug paraphernalia when injecting with sterile needles and syringes (see Additional file 2).

### HIV and HCV antibody prevalence and related factors – bio-behavioural study

The overall prevalence of antibodies for HIV was 18% (95% [CI] confidence interval 9.2% to 27%) and 59% (95% CI 49% to 69%) for HCV. Prevalence of HIV and HCV antibodies among those less than 25 years of age was 8.8% (95% CI 0.0% to 20%) and 50% (95% CI 39% to 62%), respectively. Among new injectors, the corresponding values were 3.2% (95% CI 0.0% to 7.3%) and 43% (95% CI 28% to 58%), respectively. The proportion of participants positive for both HIV and HCV antibodies was 17% (95% CI 8.5% to 25%). Both HIV and HCV prevalence differed significantly across regions. In multivariable models accounting for socio-demographic factors, duration of injecting drug history and needle/equipment sharing practices, the univariable and adjusted ORs with respect to the region with the lowest prevalence varied up to 18.4 and 12.6 for HIV and 2.8 and 3.8 for HCV, respectively.

Table 1 summarizes the analysis of factors associated with HIV and HCV prevalence in the bio-behavioural study. Separate models were built for HIV and HCV prevalence, and all factors included in the final models are presented. Due to missing values in one or more of the explanatory variables, a total of 115 (15%) cases were excluded from the multivariable analysis in addition to aforementioned 13 respondents for whom the laboratory result for either HIV or HCV was not valid. Significantly higher seroprevalence was noted among socially disadvantaged PWID; for those not working or in school, the adjusted OR for HIV antibodies was 3.3 (95% CI 1.5 to 7.0) and for HCV, 2.9 (95% CI 1.8 to 4.7). Risk of seroprevalence was also associated with a higher exposure to injecting drugs, such as the length of injecting drug use (adjusted OR for >5 years vs. <2 years: HIV 12, 95% CI 3.6 to 43; HCV 3.3, 95% CI 1.8 to 6.0; Table 1).

**Table 1:** **Individual level factors associated with HIV and HCV infections in bio-behavioural study, Poland 2004 to2005**

					**HIV**		**HCV**	
		**N**	**HIV+ (prevalence, %)**	**HCV+ (prevalence, %)**	AOR* (95% CI)	**p-value**	AOR** (95% CI)	**p-value**
Total		763	137 (18.0)	448 (58.7)				
Region	Lubelskie	87	25 (28.7)	38 (43.7)	2.65 (1.04-6.7)	0.0004	0.44 (0.19-1)	0.0176
	Lubuskie	156	14 (9.0)	86 (55.1)	1.98 (0.7-5.63)		2.04 (0.91-4.58)	
	Śląskie	60	8 (13.3)	41 (68.3)	0.71 (0.27-1.9)		1.39 (0.59-3.3)	
	Warmińsko-mazurskie	82	2 (2.4)	49 (59.8)	0.22 (0.04-1.13)		1.19 (0.49-2.89)	
	Dolnośląskie	200	32 (16.0)	120 (60.0)	2.78 (1.44-5.39)		0.73 (0.4-1.32)	
	Mazowieckie	178	56 (31.5)	114 (64.0)	ref.		ref.	
Recruitment site	Street/low-threshold	507	111 (21.9)	300 (59.2)	3.8 (1.75-8.25)	0.001	1.4 (0.76-2.59)	0.285
	Drug treatment centre	256	26 (10.2)	148 (57.8)	ref.		ref.	
Social welfare/pension as current main source of income	Yes	241	82 (34.0)	179 (74.3)	1.95 (1.17-3.26)	0.010	1.32 (0.81-2.16)	0.263
	No	509	55 (10.8)	264 (51.9)	ref.		ref.	
Currently working or in school	No	488	124 (25.4)	343 (70.3)	3.27 (1.52-7.03)	0.002	2.9 (1.79-4.68)	<0.0001
	Yes	261	12 (5.0)	100 (38.3)	ref.		ref.	
Homelessness	In last 12 months	149	58 (38.9)	110 (73.8)	2.56 (1.43-4.58)	0.0062	1.07 (0.63-1.82)	0.3310
	Before last 12 months	127	29 (22.8)	91 (71.7)	1.74 (0.91-3.32)		1.54 (0.87-2.71)	
	Never	474	50 (10.5)	242 (51.1)	ref.		ref.	
Imprisonments	In last 12 months	158	41 (26.0)	118 (74.7)	2.26 (1.18-4.36)	0.0482	2.4 (1.29-4.47)	0.0223
	Before last 12 months	183	45 (24.6)	128 (70.0)	1.27 (0.71-2.28)		1.25 (0.77-2.03)	
	Never	409	51 (12.5)	197 (48.2)	ref.		ref.	
First injection	2 to 5 years ago	215	19 (8.8)	110 (51.2)	4.03 (1.09-14.9)	<0.0001	1.24 (0.69-2.21)	<0.0001
	>5 years ago	369	113 (30.6)	268 (72.6)	12.37 (3.59-42.64)		3.3 (1.82-5.96)	
	<2 years ago	124	4 (3.2)	53 (42.7)	ref.		ref.	
Periods of everyday injecting	Yes	620	132 (21.3)	421 (67.9)	1.69 (0.45-6.3)	0.436	3.98 (1.74-9.15)	0.001
	No	113	5 (4.4)	18 (15.9)	ref.		ref.	
Sharing needles/syringes	Shared but disinfected in >1/2 such occasions	75	9 (12.0)	46 (61.3)	0.64 (0.28-1.47)	0.0132	0.69 (0.38-1.26)	0.0406
	Shared and disinfected in < =1/2 such occasions	337	85 (25.2)	240 (71.2)	1.79 (1.02-3.16)		1.4 (0.88-2.24)	
	Never shared	270	36 (13.3)	127 (47.0)	ref.		ref.	
Used opioids last 30 days	Yes	569	116 (20.4)	383 (67.3)	0.83 (0.35-1.93)	0.657	2.62 (1.45-4.71)	0.001
	No	194	21 (10.8)	65 (33.5)	ref.		ref.	
Injected last 30 days	Yes	599	126 (21.0)	406 (67.8)	1.4 (0.46-4.27)	0.555	5.02 (2.55-9.89)	<0.0001
	No	164	11 (6.7)	42 (25.6)	ref.		ref.	

*odds ratio from multivariable logistic regression model including the factors listed in the table with HIV status as the outcome variable.

**odds ratio from multivariable logistic regression model including the factors listed in the table with HCV status as the outcome variable.

Abbreviations: AOR, adjusted odds ratio; CI, confidence interval; HCV, hepatitis C virus; HIV, human immunodeficiency virus.

Of sexual exposure variables, having a PWID partner was the only one associated with HIV and HCV antibody status, but only in the univariable analysis (see Additional file 2).

### Differences by region and study site – bio-behavioural study

The prevalence of HIV varied more than tenfold by region (from 2.4% to 31.5%), whereas the overall prevalence of HCV was more consistent. However, even for HCV, important regional differences (more than twofold) were apparent among new PWID, with prevalence of HCV ranging from 25% to 56% (data not shown).

The demographics of participants included at each study site differed; the proportion females ranged from 13% to 51%, long-term injectors from 37% to 94% and opioid users from 21% to 100% (Additional file 3). Remarkably, in 4 of the 15 sites, about 60% of respondents were short-term injectors (≤5 years since first injection). Recent needle/syringe sharing (past 12 months) ranged between 9.1% and 42%. HIV and HCV seroprevalence varied between 0.0% and 47% and between 16% and 100%, respectively. In 4 of the 15 sites, a higher HCV seroprevalence (>50%) coincided with a lower HIV seroprevalence (<5%) suggesting a potential for future increases in HIV transmission at these sites [23].

### HIV case-based surveillance data 2000–2012

A total of 9,242 newly diagnosed HIV cases among individuals 15 to 44 years of age were registered from 2000 to 2012 (average annual rate 5.0 cases per 100,000). For 5,613 (61%) of these cases, the transmission category was not reported, with a range of 44% to 79% in the various regions. During this period 1,869 cases of HIV were attributed to injecting drug use; this number rose to 4,358 (95% CI, 3,936 to 4,780), when imputing missing values (Additional file 4).

### Comparison of bio-behavioural study and case-base surveillance results

**Figure 1:**
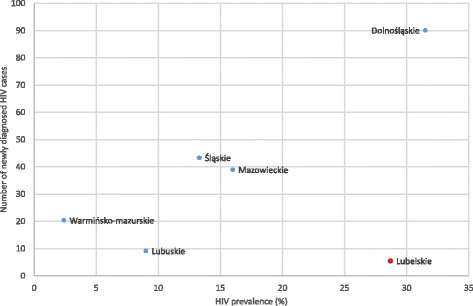
**Newly diagnosed HIV cases (2004) and HIV prevalence (2004/2005) among PWID in Poland, by region.** Numbers of newly diagnosed cases represent the estimated number of newly diagnosed cased based on multiple imputations.

**Figure 2:**
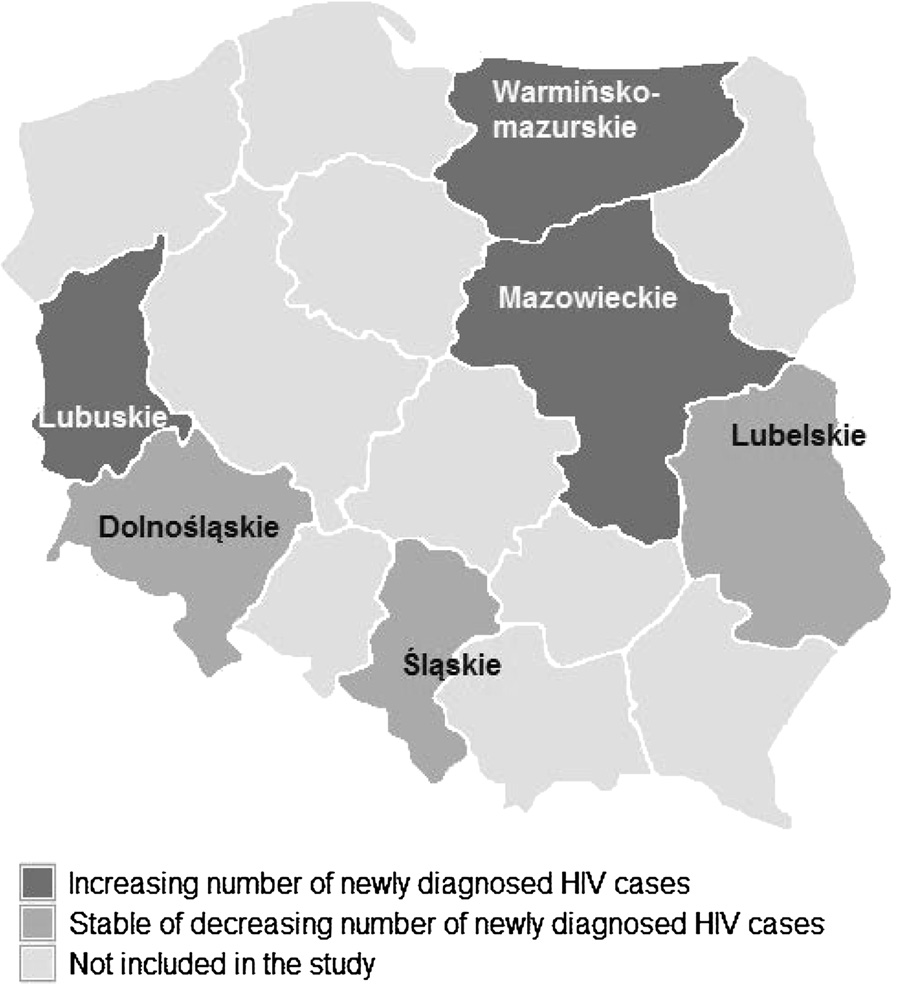
**Trends in newly diagnosed HIV cases among PWID in Poland, 2006 to 2007 vs. 2004 to 2005, by region.** Numbers of newly diagnosed cases represent estimated number of newly diagnosed cases based on multiple imputations. The increases refer to a 20% or more difference; 2004 to 2005 is the time of the bio-behavioural study.

The number of newly diagnosed HIV infections among PWID in 2004 varied by region from 6 to 90 cases, with an HIV prevalence ranging from 2.4% to 31.5%. Strong correlation existed between HIV prevalence in the bio-behavioural study and the number of newly diagnosed infections in the six regions in 2004, with the exception of the Lubelskie region (correlation coefficient [r], excluding Lubelskie, 0.93, p = 0.0214; Figure 1, Table 2). In this region a high prevalence of HIV coincided with a low number of newly diagnosed PWID-related HIV cases. From 2006 to 2011, increases of 20% or more in the annual number of reported newly diagnosed HIV cases occurred in the regions of Lubuskie, Warmińsko-mazurskie and Mazowieckie, whereas the numbers showed a clear decrease in Dolnośląskie and Śląskie (Table 2, Figure 2). In the regions where the increases occurred, the proportion of short-term injectors had previously (2004 to 2005) been >50% (all three regions) and a low HIV prevalence (<5%) had co-existed with high HCV prevalence (>50%) (Lubuskie and Warmińsko-mazurskie regions). Comparing trends from 2004 to 2007 (immediately after the bio-behavioural study was conducted), there was a significant association between the increase and the previous high (>50%) proportion of short-term injectors (p = 0.02), whereas the association with the indicator of low HIV prevalence (<5%) with high HCV prevalence (>50%) was borderline significant (p = 0.07).

**Table 2:** Comparison of HIV and HCV prevalence (2004/2005) and the trends in newly diagnosed HIV infections (2004 to 2011) in surveillance data*, in Poland by region

**Source of information**	**Indicator**	**Region**					
		**Lubelskie**	**Lubuskie**	**Śląskie**	**Warmińsko-mazurskie**	**Mazowieckie**	**Dolnośląskie**
	**Region total population (2012, Central Statistics Office)**	**2165651**	**1023317**	**4615870**	**1450697**	**5301760**	**2914362**
Bio-behavioural study	HIV prevalence (%)	28.7	9.0	13.3	2.4	16.0	31.5
	HCV prevalence (%)	43.7	55.1	68.3	59.8	60.0	64.0
	In at least one site in the region the HCV prevalence was >50% and HIV prevalence was <5%	No	Yes	No	Yes	No	No
	In at least one site in the region there was no needle/syringe programme in the city	Yes	Yes	Yes	No	No	No
	Proportion of short-term injectors (≤5 years, %)	26.7	57.1	38.3	55.4	62.7	33.5
	Proportion current (past 30 days) opioid users (%)	48.3	69.2	75.0	54.9	90	83.7
	Proportion sharing needles/syringes (past 12 months, %)	19.4	26.9	28.3	29.3	25.8	27.8
Case-based surveillance	Estimated number of new HIV diagnoses in 2004 (95% CI)*	6 (1–10)	9 (2–17)	43 (35–52)	20 (17–24)	39 (17–61)	90 (77–103)
	Peak year in 2000 to 2012; number during the peak year*	2000; 15	2009; 18	2001; 55	2000; 41	2007; 58	2003; 107
	Diagnosis rate ratios**:						
	2004 to 2005 (cross-sectional study period)	ref.	ref.	ref.	ref.	ref.	ref.
	2008 to 2009	0.7 (0.3-1.8)	1.6 (0.9-2.9)	0.4 (0.3-0.6)	0.7 (0.4-1.1)	1 (0.8-1.4)	0,7 (0,5-0,9)
	2010 to 2011	0.5 (0.2-1.5)	0.7 (0.4-1.5)	0.6 (0.4-0.8)	0.5 (0.3-0.8)	0.5 (0.4-0.8)	0,5 (0,4-0,6)

*numbers represent estimated number of newly diagnosed cased based on multiple imputations.

**rate ratios from univariable Poisson regression applied to imputed data, relating the count (outcome variable) to time period (explanatory variable).

Abbreviations: CI, confidence interval; HCV, hepatitis C virus; HIV, human immunodeficiency virus.

## Discussion

Our study found consistent results between the case-based surveillance and prevalence data on HIV infection among PWID in Poland. This is apparent both in the geographic distribution of HIV among PWID and in the association between HCV prevalence and the proportion of new injectors and the subsequent trends in newly diagnosed cases of HIV infection. While our data suggest a clear need for further investment into harm reduction services in Poland, this need seems to vary between different regions of the country, with three out of six regions apparently having a higher unmet need for the prevention of HIV and HCV infections in PWID.

We found a strong regional variation in both HIV and HCV prevalence as well as in the characteristics of the respondents (e.g., proportion of recent users, preferred substances and needle sharing behaviour) with a range in the prevalence of HIV from 2.4% to 32% and from 44% to 68% for HCV. Also, the number of newly diagnosed cases of HIV (range, 6 to 90 cases in 2004 for the different regions) during the time our study was conducted correlated well with the prevalence of HIV infection, likely validating the geographical patterns observed in the case-based surveillance study that continued until 2011. These data suggest the existence of areas in Poland where the PWID population is especially highly affected by blood borne infections. Consequently, more attention needs to be given to the geographic distribution of services.

The geographic coverage of harm reduction services seems to be persistently low. In 2011 needle and syringe programmes were available in no more than 9 of 908 cities (7 of 38 in cities with over 100,000 inhabitants) and in 8 of 16 regions in Poland. In 5 of the 15 low-threshold study sites (Lublin, Gorzów Wielkopolski, Zielona Góra, Chorzów, Sosnowiec), where we observed a substantial degree of needle sharing, there were no such programmes [20]. Similarly, clinics offering opioid substitution treatment were available in 11 of the 16 regions in Poland, which will likely decrease participation in other regions because of the need to travel to these sites [32].

A marked geographical variation in HIV prevalence has also been reported in other countries [25,26,33]. To a certain extent those differences may be attributed to the earlier history of the HIV epidemic. For example, among regions included in our study, early outbreaks of HIV (1989 to 1993) were documented in four regions (Dolnośląskie, Mazowieckie, Śląskie and Lubelskie), where we still note a high prevalence of HIV infection and potentially ongoing transmission (i.e. a high number of new HIV diagnoses). Certain characteristics may also signal the risk for future outbreaks or increases in the incidence of HIV, changes which should be detectable through case-based surveillance.

In our study subsequent HIV case-based surveillance data showed an overall decreasing trend in infection. Although no large outbreaks have taken place, transient increases in newly reported diagnosed cases occurred in three of the six studied regions (Lubuskie, Warmińsko-mazurskie and Mazowieckie) from 2006 to 2009, suggesting temporary increases in HIV transmission. These regions should be prioritised for enhancement of preventive activities and targeted for the next bio-behavioural survey.

In two of the three regions where the increases in reported HIV cases occurred, we identified sites where a high prevalence of HCV had previously (2004 to 2005) coincided with a lower prevalence of HIV. This is consistent with prior modelling studies, which suggest that a low level of HIV infection coupled with a high prevalence of HCV may indicate a potential for future HIV outbreaks [25,26] and which is likely to be indicative of frequent sharing of needles and other equipment as we found in our study.

Moreover, the increases in the number of reported HIV cases occurred in the three regions where we documented a substantial proportion (>50%) of PWID with a relatively short duration of injecting drug use (≤5 years). The combination of a higher rate of recruitment of new injectors and poor harm reduction coverage, especially among younger PWID, may contribute to the continuing spread of HIV.

The high prevalence of HIV and HCV in our study among new injectors (HIV 3.2% and HCV 42.7% among PWID with <2 years of injecting drug use) and in users less than 25 years of age (HIV 8.8% and HCV 49.5%) again strongly suggest ongoing transmission of both infections in this population. Apart from the low availability of programmes, the needs of young PWID may not be properly addressed by the existing harm reduction programmes. A study among needle and syringe programmes in Poland showed that in 2008, the average age of clients was 33 years [32], almost 5 years higher than the average age in our sample (28.5 years). This finding suggests that younger PWID may not be using these services. This calls for more intensive prevention in PWID, especially targeting young users and those injectors that started drug use recently, and with an increased emphasis on the prevention of injecting risks and HCV infection [34-39].

We confirmed a considerable degree of injecting risk behaviours among PWID in Poland. Of all study respondents, 27% admitted having shared needles and/or syringes within the 12 months prior to the study (receptive sharing). In countries with large-scale harm reduction programmes (e.g., Australia, Netherlands, some programmes in the United States), sharing of needles and other equipment can be decreased to much lower levels [40-42]. Importantly, we found high levels of equipment sharing despite evidence that the supply of sterile needles and syringes through pharmacies and through needle and syringe programmes is stable and may even be declining, possible due either to low participation by PWID or a declining need for sterile needles and syringes [20]. However, in light of our findings, this decline could also indicate that the existing programmes are progressively less able to meet the needs of PWID [21]. As noted above, young users appear not to be using these services and the geographic coverage of the services is likely insufficient.

Strict anti-drug regulations in Poland, including punishment by law for the possession of any amount of drugs, may put drug users at a social disadvantage, potentially increasing the frequency of high-risk injecting behaviours [43-45]. We found a strong association between low socioeconomic status and a history of imprisonment with higher prevalence of blood borne infections. Further analysis is needed regarding the potential effects of drug policies in Poland on the spread of blood borne infections and the effectiveness of harm reduction programmes.

Important regional and local differences in trends of newly reported diagnosed HIV cases, the prevalence of HIV and HCV infections and characteristics of PWID point to the necessity of adapting public health monitoring and responses on a local level. Decentralized availability of services and diversification of programmes offered according to local needs should be considered for PWID in Poland. Programmes should specifically include socially disadvantaged subgroups of PWID, as both HIV and HCV infections tend to concentrate in these groups; in addition specific interventions should be available, focused on reaching younger PWID. Importantly, interventions should not be limited to areas with a high prevalence of HIV. Based on our findings, high levels of risk behaviour and high rates of HCV infection existed in all regions and in some cases preceded increases in reported cases of HIV. Implementing periodic bio-behavioural prevalence studies should be integrated with an increase in harm reduction services in Poland.

There are several limitations to our findings, including the use of a convenience sample of PWID for the prevalence study. However, we used data from a survey that used exhaustive sampling for inpatient settings, and we were able to reach those PWID who were not clients of a drug treatment service by using a snow-ball method. To our knowledge there have been no other reported bio-behavioural prevalence surveys over the past decade in Poland that targeted both in-treatment and street-recruited PWID populations. Despite its limitations, our study may be the most representative bio-behavioural study among PWID in Poland to date. Additionally, the low quality of reporting of newly diagnosed HIV cases, especially the large percentage of missing data, may have compromised the trend analysis. Therefore, we performed a multiple imputation procedure to minimize possible bias due to differential reporting quality. The high correlation with prevalence data in our study supports the validity of the findings from the case-based surveillance data, but the certainty in the trend analysis may be overstated, as the uncertainty arising from multiple imputations was not accounted for. Importantly, the trends noted in the surveillance data may be influenced by the testing patterns among PWID. Testing policies did not change over the time period studied. Policies include self-initiated testing and recommended provider-initiated testing, mostly done at stationary drug treatment centres, including opioid substitution therapy centres, as well as in prisons. However, actual data on testing are limited to the number of tests performed annually, not allowing a thorough analysis of the impact of testing on the observed numbers of newly diagnosed cases of HIV. These data indicate a significant decrease in the number of tests reported among PWID [20,21]. In effect, these significant decreases in the reported number of tests may, at least partly, explain the decreasing trends in rates of newly diagnosed HIV cases among PWID and suggest even more strongly that the increases observed in some regions are of particular concern.

## Conclusions

Analysing bio-behavioural study and case-based surveillance data in parallel provided a better understanding of the epidemiology of HIV and HCV infections among PWID in Poland. Bio-behavioural and case-based data were consistent with respect to the regional distribution of HIV infection and also provided complementary information, with the proportion of new injectors and the high HCV prevalence predicting increases in HIV case rates. We identified three regions that appear to be at increased need for preventive programmes. The data point to the need for a stronger investment in harm reduction programmes in Poland.

## Additional files

Additional file 1:
**HIV and HCV prevalence among PWID by socio-demographic characteristics, Poland 2004 to 2005.** Prevalence of HIV and HCV as well as univariable associations of the prevalence with socio-demographic factors included in the bio-behavioural survey (2004 to 2005) are presented. Additional file 2:
**HIV and HCV prevalence among PWID in relation to indicators of drug use and sex-related risk, Poland 2004 to 2005.** Prevalence of HIV and HCV as well as univariable associations of the prevalence with drug use and sex-related risks included in the bio-behavioural survey (2004 to 2005) are presented. Additional file 3:
**Characteristics of the bio-behavioural study population among PWID by study site, Poland 2004 to 2005.** This file provides the breakdown of selected PWID population characteristic along with HIV and HCV prevalence rates in the regions included in the bio-behavioural study by specific study site. Additional file 4:
**Summary of the population size and the raw and imputed HIV surveillance data, by region, Poland 2000 to 2012.** This file provides tentative estimates of the regional population sizes of PWID and summarises reported new HIV diagnoses; raw data and with imputed risk group variable.

## Abbreviations

PWID: People who inject drugs
HIV: Human immunodeficiency virus
HCV: Hepatitis C virus
