# Supplementary material for: High regional variability of HIV, HCV and injecting risks among people who inject drugs in Poland: comparing a cross-sectional bio-behavioural study with case-based surveillance
Source: BMC Infect Dis. 2015 Feb 21;15:83. doi: 10.1186/s12879-015-0828-9 (PMC4340100; doi:10.1186/s12879-015-0828-9)
Supplement: Additional file 1: — HIV and HCV prevalence among PWID by socio-demographic characteristics, Poland 2004 to 2005. Prevalence of HIV and HCV as well as univariable associations of the prevalence with socio-demographic factors included in the bio-behavioural survey (2004 to 2005) are presented. [file 12879_2015_828_MOESM1_ESM.pdf]

High regional variability of HIV, HCV and injecting risks among people who inject drugs in Poland: comparing a cross-sectional bio-behavioural study with case-based surveillance

Additional file 1. HIV and HCV prevalence among PWID by socio-demographic characteristics, Poland 2004-2005.

		Total*		HIV				HCV			
		N	%	N pos.	Prev. (%)	OR (95% CI)	P-value	N pos.	Prev. (%)	OR (95% CI)	P-value
Recruitment setting	Street/low-threshold	507	66.5%	111	21.9	2.48 (1.57-3.92)	<0.0001	300	59.2%	1.06 (0.78-1.43)	0.7190
	In-patient treatment	256	33.5%	26	10.2	Ref.		148	57.8%	Ref.	
Region	Lubelskie	87	11.4%	25	28.7	0.88 (0.23-3.34)	0.0098	38	43.7	0.44 (0.14-1.36)	0.6264
	Lubuskie	156	20.5%	14	9.0	0.21 (0.08-0.57)		86	55.1	0.69 (0.21-2.26)	
	Śląskie	60	7.9%	8	13.3	0.34 (0.19-0.59)		41	68.3	1.21 (0.4-3.67)	
	Warmińsko-mazurskie	82	10.8%	2	2.4	0.05 (0.03-0.12)		49	59.8	0.83 (0.37-1.89)	
	Mazowieckie	200	26.2%	32	16.0	0.41 (0.26-0.66)		120	60.0	0.84 (0.56-1.27)	
	Dolnośląskie	178	23.3%	56	31.5	Ref.		114	64.0	0.44 (0.14-1.36)	
Sex	Male	540	71.3%	99	18.4	1.06 (0.76 -1.47)	0.7175	328	61.1	1.38 (0.93 -2.04)	0.1236
	Female	217	28.7%	38	17.5	Ref.		116	53.5	Ref.	
Age group	<25	295	38.6%	26	8.8	Ref.	0.0071	145	49.3	Ref.	0.0015
	25-34	304	39.7%	60	19.7	2.55 (1.03-6.30)		184	60.5	1.57 (1.00-2.46	
	35-44	127	16.6%	42	33.6	5.25 (1.38-20.03)		89	71.2	2.58 (1.42-4.69)	
	45+	39	5.1%	9	23.1	3.12 (0.44-21.93)		30	76.9	3.40 (1.65-7.00)	

		Total		HIV				HCV			
		N	%	N pos.	Prev. (%)	OR (95% CI)	P-value	N pos.	Prev. (%)	OR (95% CI)	P-value
Completed education	Elementary school (8 years)	229	30.4%	55	24.1	Ref.	0.0998	156	68.4	Ref.	<0.0001
	Vocational (12 years)	225	29.9%	44	19.6	0.76 (0.48-1.22)		138	61.3	0.73 (0.52-1.04)	
	High school (12 years)	230	30.5%	34	14.9	0.55 (0.20-1.51)		124	54.4	0.55 (0.37-0.82)	
	Bachelor/ higher (15/17 years)	69	9.2%	4	5.8	0.19 (0.04-1.03)		25	36.2	0.26 (0.13-0.53)	
Present employment	Full-time or part-time	193	25.8%	10	5.2	0.17 (0.06 – 0.51)	<0.0001	75	38.9	0.29 (0.21-0.39)	<0.0001
	Not working (house-keeping, retirement etc.)**	100	13.4%	38	38.0	1.90 (0.68-5.30)		83	83.0	2.20 (1.15-4.20)	
	Unemployed	348	46.5%	85	24.4	Ref.		240	69.0	Ref.	
	Student	68	9.1%	3	4.4	0.14 (0.05 – 0.41)		25	36.8	0.26 (0.18-0.38)	
	Other status	40	5.3%	1	2.5	0.08 (0.001 – 0.88)		20	50.0	0.45 (0.11-1.85)	
Present source of income	Stable (work of study participant or family member)	359	47.9%	23	6.4	Ref.	<0.0001	170	47.4	Ref.	0.0001
	Savings/ stipend	20	2.7%	3	15.0	2.59 (0.22-30.53)		11	55.0	1.35 (0.62-2.94)	
	Pension	78	10.4%	29	37.2	8.67 (2.54-29.63)		61	78.2	4.03 (2.21-7.36)	
	Social welfare or none	163	21.7%	53	32.5	7.00 (3.31-14.80)		118	72.4	2.92 (1.71-4.99)	
	Other	130	17.3%	29	22.3	4.21 (1.56-11.35)		83	63.8	1.95 (0.81-4.69)	

		Total		HIV				HCV			
		N	%	N pos.	Prev. (%)	OR (95% CI)	P-value	N pos.	Prev. (%)	OR (95% CI)	P-value
Homelessness	In last 12 months	149	19.9%	58	38.9	5.42 (3.39-8.66)	<0.0001	110	73.8	2.74 (1.83-4.10)	<0.0001
	Before last 12 months	127	16.9%	29	22.8	2.49 (1.85-3.36)		91	71.7	2.41 (1.63-3.57)	
	Never	474	63.2%	50	10.5	Ref.		242	51.1	Ref.	
Imprisonment	Ever	341	45.5%	86	25.2	2.37 (1.61-3.47)	<0.0001	246	72.1	2.79 (2.05-3.78)	<0.0001
	Never	409	54.5%	51	12.5	Ref.		197	48.2	Ref.	
Dependency treatment	In last 12 months	496	53.2%	54	13.6	1.16 (0.63-2.13)	<0.0001	242	61.1	3.00 (1.97-4.56)	<0.0001
	Before last 12 months	224	30.1%	68	30.4	3.20 (1.74-5.88)		156	69.6	4.37 (2.74-6.97)	
	Never	125	16.8%	15	12.0%	Ref.		43	34.4	Ref.	

*“missing” category not presented; **includes persons not working because of health reasons;
