# Supplementary material for: High regional variability of HIV, HCV and injecting risks among people who inject drugs in Poland: comparing a cross-sectional bio-behavioural study with case-based surveillance
Source: BMC Infect Dis. 2015 Feb 21;15:83. doi: 10.1186/s12879-015-0828-9 (PMC4340100; doi:10.1186/s12879-015-0828-9)
Supplement: Additional file 2: — HIV and HCV prevalence among PWID in relation to indicators of drug use and sex-related risk, Poland 2004 to 2005. Prevalence of HIV and HCV as well as univariable associations of the prevalence with drug use and sex-related risks included in the bio-behavioural survey (2004 to 2005) are presented. [file 12879_2015_828_MOESM2_ESM.pdf]

High regional variability of HIV, HCV and injecting risks among people who inject drugs in Poland: comparing a cross-sectional bio-behavioural study with case-based surveillance

Additional file 2. HIV and HCV prevalence among PWID in relation to indicators of drug use and sex related risk, Poland 2004-2005.

		Total[*]		HIV				HCV			
		N	%	N pos.	Prev. (%)	OR (95% CI)	P-value	N pos.	Prev. (%)	OR (95% CI)	P-value
First injection	<2 years prior to study	124	17.5%	4	3.2	Ref.	<0.0001	53	42.7	Ref.	<0.0001
	2 to 5 years prior to study	215	30.4%	19	8.8	2.92 (1.07-7.95)		110	51.2	1.38 (0.98-1.93)	
	>5 years prior to study	369	52.1%	113	30.6	13.34 (3.09-57.61)		268	72.6	3.52 (1.90-6.51)	
Last Injection	In last 30 days	599	82.3%	126	21.0	2.88 (0.95-8.68)	0.0695	406	67.8	5.47 (3.37-8.89)	<0.0001
	Before last 30 days	129	17.7%	11	8.5	Ref.		36	27.9	Ref.	
	Unknown	35			0.0			6	17.1		
Periods of every day injecting	Ever	620	84.6%	132	21.3	5.82 (2.42-14.02)	<0.0001	421	67.9	11.24 (6.33-19.98)	<0.0001
	Never	113	15.4%	5	4.4	Ref.		18	15.9	Ref.	
Current drugs	Opioid users	569	74.6%	116	20.4	2.30 (1.00-5.31)	0.1512	383	67.3	5.51 (2.95-10.32)	<0.0001
	Stimulant users not using opioids	139	18.2%	14	10.1	Ref.		38	27.3	Ref.	
	Other	9	1.2%	1	11.1	1.13 (0.19-6.86)		4	44.4	2.13 (1.01-4.47)	
	Unknown	46	6.0%	6	13.0	1.58 (0.68-3.65)		23	50.0	2.66 (1.08-6.54)	
Ever sharing needles/syringes	Yes	452	62.60	100	22.1	1.85 (1.20-2.84)	0.0138	303	67.0	2.29 (1.37-3.81)	0.0063
	No	270	37.4%	36	13.3	Ref.		127	47.0	Ref.	

		Total		HIV				HCV			<0.0001
		N	%	N pos.	Prev. (%)	OR (95% CI)	P-value	N pos.	Prev. (%)	OR (95% CI)	P-value
Sharing other equipment when injecting with clean needles	>1/2 of such injections	137	22.8%	35	25.5	1.47 (0.71-3.04)	0.3391	244	67.9	1.29 (0.77-2.14)	0.4302
	≤1/2 of such injections	464	77.2%	87	18.8	Ref.		176	62.9	Ref.	
Sex work	Never	688	92.47	127	18.45	Ref.	0.9289	402	58.43	Ref.	0.4001
	Ever	56	7.53	10	17.85	0.97 (0.40-2.35)		37	66.07	1.39 (0.65-3.00)	
Condom use	Never or rarely	374	50.20	50	13.37	0.30 (0.21-0.42)	<0.001	217	58.02	0.67 (0.42-1.08)	0.0565
	Frequently	230	30.87	39	16.96	0.40 (0.23-0.69)		128	55.65	0.61(0.40-0.92)	
	Always	141	18.92	48	34.04	Ref.		95	67.38	Ref.	
IDU sex partner	In last 12 months	208	27.92	53	25.48	3.18 (1.32-7.70)	0.0360	148	71.15	2.76 (1.78-4.29)	<0.0001
	Before last 12 months	238	31.94	55	23.11	2.77 (1.07-7.16)		151	63.45	1.95 (1.30-2.92)	
	Never	299	40.13	29	9.70	Ref.		141	47.16	Ref.	
History of sexually transmitted infection	Never	665	95.14	119	17.89	Ref.	0.1158	388	58.34	Ref.	0.3688
	Ever	34	4.86	11	32.35	2.20 (0.82-5.87)		17	50.00	0.71 (0.34-1.49)	

* “missing” category not presented; **includes those that have never shared needless;
