# Supplementary material for: High regional variability of HIV, HCV and injecting risks among people who inject drugs in Poland: comparing a cross-sectional bio-behavioural study with case-based surveillance
Source: BMC Infect Dis. 2015 Feb 21;15:83. doi: 10.1186/s12879-015-0828-9 (PMC4340100; doi:10.1186/s12879-015-0828-9)
Supplement: Additional file 3: — Characteristics of the bio-behavioural study population among PWID by study site, Poland 2004 to 2005. This file provides the breakdown of selected PWID population characteristic along with HIV and HCV prevalence rates in the regions included in the bio-behavioural study by specific study site. [file 12879_2015_828_MOESM3_ESM.pdf]

High regional variability of HIV, HCV and injecting risks among people who inject drugs in Poland: comparing a cross-sectional bio-behavioural study with case-based surveillance

Additional file 3. Characteristics of the bio-behavioural study population among PWID by study site, Poland 2004-2005.

Region	City/site	Recruitment settings (N)				Age	Females	>5 years since first injection	Using opiates past 30 days	Sharing needles/syringes last 12 months	HIV (+)	HCV (+)
		DX*	DF	OT	LT	(mean)	(N, %)	(N, %)	(N, %)	(N,%)	(N, %)	(N,%)
Lubelskie	Lublin	30	-	-	12	32.1	9, 27.2%	23, 79.3%	26, 63.4%	6, 20.7%	4, 9.7%	10, 24.4%
	Puławy	-	-	-	50	30.9	20, 40.8%	32, 69.6%	16, 34.8%	8, 18.6%	21, 45.6%	28, 60.9%
	TOTAL	30	-	-	62	31.2	29, 35.9%	55, 73.3%	42, 48.3%	14, 19.4%	25, 28.7%	38, 43.7%
Lubuskie	Cibórz	-	87	-	-	27.9	21, 24.1%	34, 42.5%	76, 92.7%	19, 24.1%	12, 14.6%	58, 70.7%
	Gorzów Wlkp.	-	-	26	12	23.9	12, 31.5%	9, 40.9%	8, 21.0%	5, 14.3%	2, 5.4%	6, 16.2%
	Nowy Dworek	-	21	-	-	26.0	3, 14.2%	8, 38.0%	18, 90.0%	14, 70.0%	0, 0.0%	18, 90.0%
	Zielona Góra	-	-	-	17	24.7	9, 52.9%	6, 54.5%	6, 35.2%	1, 9.1%	0, 0.0%	4, 23.5%
	TOTAL	-	108	26	29	26.4	45, 28.2%	57, 42.9%	108, 69.2%	39, 26.9%	14, 9.0%	86, 55,1%
Śląskie	Chorzów	-	-	-	17	31.6	8, 47.0%	16, 94.1%	17, 100%	5, 29.4%	3, 17.6%	17, 100.0%
	Katowice	-	-	-	14	26.5	4, 28.5%	6, 42.8%	9, 64.2%	4, 28.6%	1, 7.1%	7, 50.0%
	Sosnowiec	-	-	-	29	28.8	11, 37.9%	15, 51.7%	19, 65.5%	8, 27.6%	4, 13.7%	17, 58.6%
	TOTAL	-	-	-	60	29.1	23, 38.3%	37, 61.2%	45, 75.0%	17, 28.3%	8, 13.3%	41, 68.3%

Region	City/site	Recruitment settings (N)				Age	Females	>5 years since first injection	Using opiates past 30 days	Sharing needles/syringes last 12 months	HIV (+)	HCV (+)
		DX*	DF	OT	LT	(mean)	(N, %)	(N, %)	(N, %)	(N,%)	(N, %)	(N,%)
Warmińsko-mazurskie	Barczewo	-	14	-	-	26.9	0, 0.0%	5, 45.4%	7, 50.0%	1, 7.7%	0, 0.0%	10, 71.4%
	Elbląg	-	23	-	-	25.8	0, 0.0%	9, 40.9%	12, 56.5%	6, 26.1%	1, 4.5%	17, 77.2%
	Olsztyn	15	-	-	31	26.5	6, 13.0%	19, 45.2%	26, 56.5%	15, 37.5%	1, 2.2%	22, 47.8%
	TOTAL	15	37	-	31	26.2	6, 7.3%	33, 44.6%	45, 54.9%	39, 26.9%	2, 2.4%	49, 59.8%
Mazowieckie	Warszawa	-	-	-	200	27.3	49, 24.5%	72, 37.3%	180, 90.0%	50, 25.8%	32, 16.0%	120, 60.0%
Dolnośląskie	Wrocław1	48	-	-	-	32.6	23, 51.1%	32, 74.4%	35, 77.7%	18, 41.9%	8, 17.8%	23, 51.1%
	Wrocław2	-	-	-	130	30.3	44, 33.0%	83, 63.8%	114, 85.7%	31, 23.3%	48, 36.1%	91, 68.4%
	TOTAL	48	-	-	130	30.8	67, 37.6%	115, 66.4%	149, 83.7%	49, 27.8%	56, 31.5%	114, 64.0%
Overall		93	145	26	512	28.5	219, 28.6%	369, 48.4%	569, 74.5%	191, 26.6%	137, 18.0%	451, 58.8%

*DX - Detoxification ward; DF – long term drug-free treatment; OT – outpatient treatment; LT – low-threshold settings including street outreach and snow-ball recruitment.
