# Supplementary material for: High regional variability of HIV, HCV and injecting risks among people who inject drugs in Poland: comparing a cross-sectional bio-behavioural study with case-based surveillance
Source: BMC Infect Dis. 2015 Feb 21;15:83. doi: 10.1186/s12879-015-0828-9 (PMC4340100; doi:10.1186/s12879-015-0828-9)
Supplement: Additional file 4: — Summary of the population size and the raw and imputed HIV surveillance data, by region, Poland 2000 to 2012. This file provides tentative estimates of the regional population sizes of PWID and summarises reported new HIV diagnoses; raw data and with imputed risk group variable. [file 12879_2015_828_MOESM4_ESM.pdf]

High regional variability of HIV, HCV and injecting risks among people who inject drugs in Poland: comparing a cross-sectional bio-behavioural study with case-based surveillance

Additional file 4. Summary of the population size and the raw and imputed HIV surveillance data, by region, Poland 2000 – 2012.

	Population size (2008)		*HIV reports in 2000 - 2012 (aged 15-44 at diagnosis)*			
Region	Total population (aged 15-44)	Tentative number of PWID (among current problem drug users)*	Total (average annual rate per 100,000)	Unknown transmission category	PWID	PWID imputed data
Dolnośląskie	1249975	1688-1986	1323 (9.6)	762	454	1118 (1055-1181)
Kujawsko-pomorskie	916379	1238-1456	325 (3.2)	196	65	163 (137-190)
Lubelskie	946310	1278-1503	224 (2.2)	122	40	103 (66-139)
Lubuskie	448112	605-712	239 (4.8)	152	54	168 (127-210)
Łódzkie	1073809	1450-1706	569 (4.8)	169	198	326 (302-350)
Małopolskie	1485514	2006-2360	413 (2.5)	307	48	154 (99-209)
Mazowieckie	2261239	3054-3593	1439 (5.8)	822	127	540 (373-707)
Opolskie	462311	624-734	180 (3.5)	127	37	121 (88-153)
Podkarpackie	956380	1292-1519	149 (1.4)	84	33	90 (68-111)
Podlaskie	529783	715-841	170 (2.9)	75	48	122 (30- .)
Pomorskie	991285	1339-1575	460 (4.2)	236	91	230 (190-269)
Śląskie	2025868	2736-3219	801 (3.6)	524	159	438 (309-566)
Świętokrzyskie	543806	734-864	83 (1.4)	61	8	45 (11-79)
Warmińsko-mazurskie	642560	868-1021	406 (5.7)	223	130	356 (325-388)
Wielkopolskie	1536465	2075-2441	482 (2.9)	259	60	176 (112-239)
Zachodniopomorskie	740438	1000-1176	353 (4.3)	213	69	202 (170-234)
Unknown	-	-	1626	1281	248	-
Total	16810234	22710-26711	9242 (5.0)	5613	1,869	4358 (3936-4780)

*Tentative number of injectors countrywide was established using the estimate for opioid injectors (17,000 – 20,000) and the proportion of PWID in our sample who injected opioids (74.5%). Subsequently the country wide injecting prevalence (0,14% - 0.16%) was applied to regions' population aged 15-44. The distribution of PWID across the country is likely to be uneven so these estimates should be treated with caution.
